# Hyperphosphatemic Tumoral Calcinosis after Total Knee Arthroplasty

**DOI:** 10.1155/2017/1528201

**Published:** 2017-10-16

**Authors:** Takeshi Mochizuki, Katsunori Ikari

**Affiliations:** ^1^Department of Orthopedic Surgery, Kamagaya General Hospital, Chiba, Japan; ^2^Institute of Rheumatology, Tokyo Women's Medical University, Tokyo, Japan

## Abstract

We report a case of hyperphosphatemic tumoral calcinosis (TC) that occurred after total knee arthroplasty. A 64-year-old Japanese man presented with painful swellings in both shoulders, the left elbow, and the right hip that developed after he underwent total knee arthroplasty (TKA). The pathology of the patient's bone at the time of TKA included a thick osteoid seam with calcareous deposition at the margin of the trabecular bone, which is not generally seen in osteoarthritis. Computed tomography scans of the swollen joints demonstrated leaflet and amorphous calcification masses around the joints. We diagnosed the patient with TC. The present case highlights that TC lesions are rare but should be considered in the differential diagnosis of subcutaneous soft and hard masses around the joint.

## 1. Introduction

Tumoral calcinosis (TC) is a distinct disease of uncertain etiology that is defined by massive extra-articular soft tissue deposition of calcium phosphate around large joints, such as the hips, knees, shoulders, elbows, and wrists [1, 2]. Secondary TC may be aroused by renal failure, hypervitaminosis D, sarcoidosis, and hyperparathyroidism, but there are no demonstrable abnormalities in calcium metabolism [3]. On the other hand, familial forms of TC related to inheritable characteristics are caused by autosomal recessive mutations of such genes as GalNAc transferase 3 (*GALNT 3*), fibroblast growth factor 23 (*FGF-23*), and *α*Klotho [4, 5].

Idiopathic TC has two subtypes. The normophosphatemic TC subtype is characterized by normal serum phosphate and calcium levels, and the hyperphosphatemic TC subtype is characterized by elevated serum phosphate levels with normal serum calcium levels [6].

The total knee arthroplasty is common surgery in orthopaedics. In the past, there was no report that TC has occurred after total knee arthroplasty. Here, we report a case of TC that occurred after total knee arthroplasty.

## 2. Case Report

**Figure 1:**
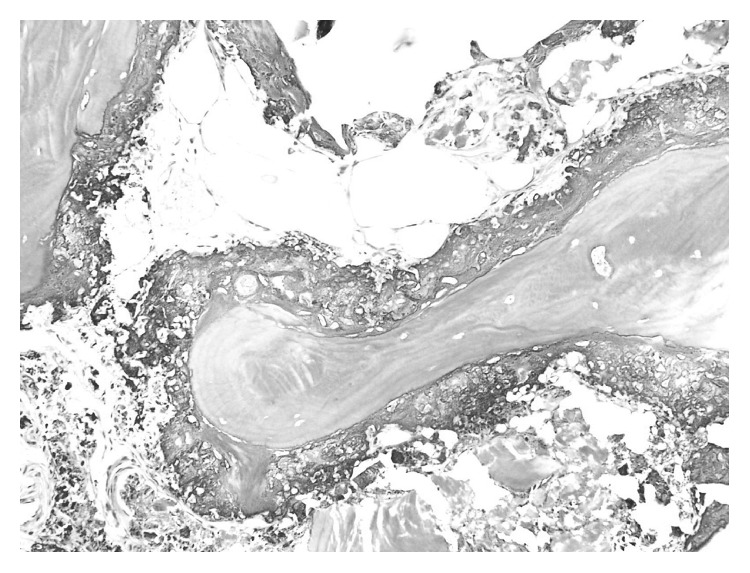
The pathology of the bone in total knee arthroplasty (hematoxylin and eosin staining, ×200).

**Figure 2:**
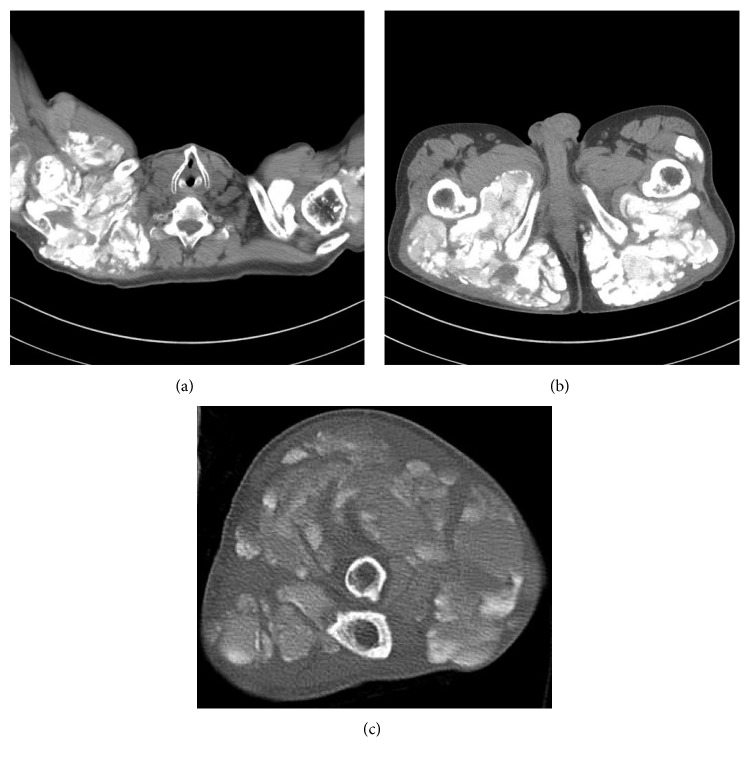
Computed tomography findings at the shoulders (a), hips (b), and left elbow (c).

A 64-year-old Japanese man presented with pain, swelling, and limited motion in both knees with gait disturbances. We diagnosed the patient with osteoarthritis and performed total knee arthroplasty in both knees. Intraoperative findings revealed that the patient's bone was very hard. The pathology of the patient's bone included a thick osteoid seam with calcareous deposition at the margin of the trabecular bone, such as is not usually seen in osteoarthritis (Figure 1()). About 1 year later, he presented with painful swellings in both shoulders, the left elbow, and the right hip. There was no history of fever, trauma, renal dysfunction, or endocrinal abnormalities, and there was no history of similar swelling in other family members. The patient's laboratory results were as follows: C reactive protein, 8.5 mg/dL; white blood cell count, 8700/*µ*L; creatinine, 0.73 mg/dL; calcium, 9.7 mg/dL; phosphate, 7.5 mg/dL (normal value: 2.9–4.9); total type I procollagen N-terminal propeptide, 605 *µ*g/L (normal value: 18.1–74.1); and tartrate-resistant acid phosphatase-5b, 6710 mU/dL (normal value: 170–590). Rheumatoid factor and anti-cyclic citrullinated peptide antibody were absent. Plain radiographs of the left shoulder disclosed a large, lobulated, and periarticular soft tissue calcareous mass. Computed tomography (CT) scans demonstrated leaflet and amorphous calcification masses around both shoulders and into the latissimus dorsi muscle and axilla, the right hip, and the left elbow with same calcification masses (Figures 2()–2()). The calcification mass on the left shoulder was punctured, and turbid, white, and chalky fluids were aspirated. The cytologic diagnosis of the fluids included bacteria and histiocytes, while the bacteriological culture of the fluids did not contain bacteria. Moreover, the incisions in the left axilla were red and took a prolonged time to heal. Eventually, the calcification mass on the left shoulder recurred and became worse and larger. We diagnosed TC from physical, radiological, and pathological findings. The patient has experienced difficulties in daily life, but he does not want aggressive treatment.

## 3. Discussion

TC is a heterogeneous disorder of obscure etiology characterized by extensive nonosseous calcification, especially in the periarticular soft tissue regions of major joints [2]. TC commonly affects the periarticular regions of the hips, shoulders, and elbows; it may rarely affect distal locations like the hands and feet [1]. In this case, TC appeared in both shoulders, both hips, and the left elbow. TC most commonly affects young people, although occurrences in all age groups ranging from infancy to old age have been reported in the literature. In two cases in middle-aged people, a 53-year-old man had rheumatoid arthritis and a 62-year-old woman had systemic sclerosis [7, 8]. Regardless of primary or secondary presentation, patients typically present with progressively increasing masses in the site of origin. Hence, the patients often report pain and limited motion around the involved joints.

The etiology of TC, including the mechanism and genetic basis of the entity, remains obscure. Recent molecular research has suggested a role of *GALNT 3* and *FGF-23* in familial TC. The novel homozygous missense mutation in exon 3 of the *GALNT 3* has been reported as one of the factors in the gene [5]. However, the phenotypes of *GALNT 3* mutation-related calcification disorders have yet to be clarified.

In our case, the patient underwent total knee arthroplasty before the onset of TC. The pathological findings of the bone at surgery indicated calcium deposition. This finding may suggest the bone of TC. We had better doubt abnormal bone metabolism including TC if there is a finding of such a bone.

The treatment of TC is considered to be the reduction of serum phosphate through a restricted diet and oral phosphate-binding drugs, such as calcium carbonate. Clinical and radiographical improvement has been reported after phosphorus deprivation therapy [9]. Surgical excision is the recommended management for removing abnormal tissue to prevent recurrence and improve limited motion [10, 11]. However, incomplete resection is associated with higher rates of recurrence [6, 12]. In the surgical removal of the TC, we must pay attention to the wound infection from our result. In chronic kidney disease patients with secondary TC, early subtotal parathyroidectomy may be an approach to treatment to avoid aggravation [13]. At present, treatment for TC has not been established.

The case has occurred TC after total knee arthroplasty. We suggested that there was no casual connection between the surgery and the onset of TC. The present case highlights that TC lesions are rare but should be considered in the differential diagnosis of subcutaneous soft and hard masses around the joint. Moreover, when a surgeon observes a hard bone during surgery, TC should be considered. Therefore, we think that the most important thing is to actively examine as CT for early diagnosis after the surgery.
